# Attitude towards Older People According to Sociodemographic and Educational Variables in Students of a Chilean University

**DOI:** 10.3390/geriatrics7060130

**Published:** 2022-11-23

**Authors:** Rodrigo Yáñez-Yáñez, Maria Antonia Parra-Rizo, Nelson McArdle-Draguicevic, Nathalie Valdés-Valdés, Gabriel A. Rojas, Leslith Gamín, Paulina Lorca, Francisca Acevedo-Carrizo, Rafael Zapata-Lamana, Caterin Diaz-Vargas, Igor Cigarroa

**Affiliations:** 1 Departamento de Kinesiologia, Universidad de Magallanes, Punta Arenas 6200000, Chile; 2 Faculty of Health Sciences, Valencian International University (VIU), 46002 Valencia, Spain; 3 Department of Health Psychology, Faculty of Social and Health Sciences, Campus of Elche, Miguel Hernandez University (UMH), 03202 Elche, Spain; 4 Escuela de Kinesiología, Facultad de Salud, Universidad Santo Tomás, Iquique 1100000, Chile; 5 Escuela de Kinesiología, Facultad de Salud, Universidad Santo Tomás, Valdivia 5090000, Chile; 6 Magister en Gerontología Clínica Interdisciplinar, Universidad Santo Tomás, Antofagasta 1240000, Chile; 7 Escuela de Terapia Ocupacional, Facultad de Salud, Universidad Santo Tomás, Viña del Mar 2520000, Chile; 8 Escuela de Educación, Universidad de Concepción, Los Ángeles 4440000, Chile; 9 Escuela de Kinesiología, Facultad de Salud, Universidad Santo Tomás, Los Ángeles 4440000, Chile; 10 Centro de investigación de Gerontología Aplicada (CIGAP), Universidad Santo Tomas, Antofagasta 1240000, Chile

**Keywords:** attitude, aging, old age, higher education, ageism, old ageism, stereotypes, prejudices, older people

## Abstract

Current evidence suggests that attitude towards older people may be associated with sociodemographic and educational variables; hence, a positive attitude towards older people is key when training new university professionals. However, there is little evidence of this association in Chilean university students. The objective was to analyze students from a Chilean university’s attitudes towards older people, according to sociodemographic and educational variables. Analytical and cross-sectional study; 515 students from a Chilean university were consulted online about their attitude towards older people using Kogan’s Attitudes towards Old People scale. Additionally, sociodemographic and educational variables were recorded. The average score for positive attitude was 70.8 (±9.7), while the negative attitude score was 68.3 (±11.6). The total score was 139.1 (±16.6). Mostly, university students perceive themselves with a low-level positive attitude (61.2%). Additionally, older university students (26–42 years old); women; Chileans; students of law, speech therapy, and occupational therapy; students in their final years of the programs; and those who had training in older people outside the university have a more positive attitude towards older people. In Conclusion, a profile of sociodemographic and educational characteristics of students with a lower and higher attitude towards older age was investigated. These results are relevant since the way of seeing the aging process could regulate the training of future professionals and consequently generate changes in dealing with older people. Young people’s perception of ageing would affect the treatment and incorporation of the older people in society and the adaptation of policies in this age group.

## 1. Introduction

Nowadays, it is well-known that the world’s population is aging, day by day and at an accelerating rate. In 2030, one in six people in the world will be sixty years old or older, increasing from 1000 million in 2020 to 1400 million. By the year 2050, the population of people in that age range will have doubled (2100 million) [1]. Consequently, studies about old age have increased.

Etymologically, the word old comes from the Latin word vetus, which comes from the Greek word etos, meaning years. In general terms, old age is measured by an accumulation of time, as time gone by [2]. It has been observed that the representation of old age is ambivalent; that is, on some occasions it is associated with experience and wisdom, while on others it is seen as a time of illness and difficulty [3].

Every society builds an image of itself that changes through time, evolving in accordance with different historical, social, political, and cultural contexts and social class, therefore adapting and fluctuating. Provided that these images are strong and stable, they turn into social stereotypes [4]. Despite the concepts of “elderly” and “old age” varying in each society, the terms usually carry negative connotations related to disabilities and biological decline, such as fragility, stiffness, and dependency, as well as positive attributes, such as wisdom, longevity, experience, and personal success [4,5].

Attitudes towards older people include feelings, cognitions, and behaviors related to the process of aging as a personal experience [6]. The term ageism was coined by Robert Butler (1969), and it refers to prejudice directed towards one group due to their age. It comprises both stereotypes and discrimination based on age [7]. Although ageism can be directed towards younger adults, it commonly refers to negative associations and behaviors directed towards older adults [7]. In this line, “Ageism against older people” is a term used to identify attitudes towards the older population, the ageing process, and old age. Specifically, this concept refers to “stereotypes, prejudices and discriminatory behavior against older people” [7]. Palmore (1999) suggests that there are at least nine major categories of negative stereotypes associated with old age: illness, impotency, ugliness, mental decline, mental illness, uselessness, isolation, poverty, and depression. Thus, older adults are often perceived to be sick, lonely, bored, living in poverty, and often irritable and angry [3]. Ageism towards older people would be determined by age norms and related to a social construct instilled from birth and varied according to age and sex of the person; in this way, the social norms to be followed by a child, an adolescent, an adult, or older people will be different from each other [8].

Stereotypes work unconsciously and are internalized through life. Older adults often incorporate these stereotypes into their beliefs and self-perception, which significantly affects their cognitive and functional well-being, aiding illness and disability, and negatively affecting their behavior [9]. Levy (2003) suggests that the development and functioning of aging self-stereotypes have identifiable characteristics: (a) they originate in the form of aging stereotypes as early as in childhood and are reinforced in adulthood; (b) aging self-stereotypes, as well as aging stereotypes, can operate below awareness; and (c) in old age, aging stereotypes become self-stereotypes [10].

Ageism has been observed to impact areas such as job satisfaction [11], cognitive functioning [12], and psychological wellbeing [13]. Levy’s (2009) Stereotype Embodiment Theory provides a framework for understanding the impact of ageism on health and well-being [9]. In that context, currently, increasing interest in the identification and analysis of attitudes towards the elderly among younger populations exists [14]. Previous studies have analyzed the stereotypes and attitudes towards older people, and they have shown that university students, professionals, and society have negative, discriminative, and judgmental attitudes towards the geriatric population [14,15].

Particularly, when attitude towards aging in university students has been analyzed, previous studies have observed a negative perception, despite differences in sex, program, or academic year [16]. For instance, Gutierrez et al. (2019) showed that university students with a master’s degree held fewer stereotypes towards old age, mainly in areas such as character, personality, and social motivation. Negative stereotypes usually associate the older population with mental rigidity and fewer vital interests [17]. Moreover, Chamorro et al. (2014) observed that students in programs related to health sciences had a more positive image than students in social sciences [18]. In terms of their academic year, a study carried out in 2018 with a sample of 200 physiotherapy and occupational therapy students concluded that freshmen had significantly more ageist stereotypes than junior or senior students [19].

Additionally, previous studies have identified educational factors that may influence attitudes towards old age, such as the type and amount of information received about older people, social distance between the observer and the elderly, and individual differences such as social class, among others [20]. Furthermore, age has proven to be an important predictor of attitudes towards older adults. To illustrate this, Chu et al. (2020) observed that younger people tend to have more negative opinions towards older adults than older adults have about themselves [20]. In addition, it is suggested that carrying out activities associated with older people or asking students to use objects typically used by older adults can foster a positive attitude towards the care of older people [21].

In this context, it is crucial to analyze university students’ attitudes towards older people, as some of them will eventually provide care to the growing elderly population [22]. Therefore, it is necessary to know their attitudes and to be able to create adequate educational programs directed towards younger people [23].

As an example, in health-programs students, one of the barriers to providing quality services to older adults is found in negative attitudes and stereotypes, which, in turn, negatively affect health care outcomes and reduce service efficiency [24]. It has been observed that the quality of health services provided to the older subjects is strongly influenced by the attitude of health workers towards them [25].

The objective of this study is to analyze students from a Chilean university’s attitudes towards older people according to sociodemographic and educational variables. Based on the existing evidence, we hypothesize that there is an association between sociodemographic and educational variables and the attitude towards older people in students from a Chilean university, with older students, female students, students on the last years of their programs, and students with training on aging presenting a more positive attitude towards aging compared to younger men, those in the first years of their programs, and those without training on issues related to aging.

## 2. Materials and Methods

### 2.1. Study Design

The study had a cross-sectional, nonexperimental, correlational–causal scope.

### 2.2. Population, Sample, and Place Where the Study Was Carried Out

Universidad Santo Tomás is a private Chilean university founded in 1988. In addition to its headquarters in Santiago, it has 12 other campuses in different regions of Chile. Currently, it has 25,161 students distributed in 30 undergraduate programs in its eight faculties: Sciences, Social Sciences and Communications, Law, Economics and Business, Education, Engineering, Natural Resources and Veterinary Medicine, and Health. This study was conducted in 11 of the 13 university campuses, and students from 13 of the 30 undergraduate programs answered the survey [26].

Students 18 years old and older from the Universidad Santo Tomás, Chile, were invited to participate in this study (11 of the 13 university campuses were included). The sample size was calculated considering the heterogeneity of 50%, a margin of alpha error of 5%, and a confidence level of 95%. A probabilistic, stratified-by-sex sample of 519 student was included. The following inclusion criteria were considered: (a) being an undergraduate student at Universidad Santo Tomás, Chile, (b) being enrolled in any program of the university, (c) in any year, (d) in Chile or abroad, (e) and being between 18 and 42 years old. A total of 4 students were not included (over the age limit).

### 2.3. Procedure

An alliance between professors in Universidad Santo Tomás was formed in order to have a representative in each campus who oversaw inviting students to participate in the study.

The invitation was made through internal university social networks for two weeks. The invitations presented a link to an online platform (Google Forms) which hosted the questionnaires used for this research. Said questionnaires were available from 4th October to 5th November 2021. The participants were given a phone number to call in case of doubts or questions. All participants signed an informed consent, before the data were collected, which had been approved by the Research Ethics Committee of the Universidad Santo Tomás (southern macrozone) (protocol code n°83-21). All procedures were carried out according to the Declaration of Helsinki and Singapore Statement.

### 2.4. Variables and Measurement Tools

*Attitude towards old age:* Attitude towards old age was measured through Kogan’s Attitudes towards Old People (KAOP) test, which consists of 34 statements regarding older people. In the questionnaire, 17 of the statements are worded positively and 17 negatively. The scale is designed from a Likert-type questionnaire, with six categories as answers: (A) Strongly disagree; (B) Slightly disagree; (C) Disagree; (D) Agree; (E) Slightly agree; (F) Strongly agree. The minimum and maximum scores were 34 and 204. A higher score reflects a positive attitude towards older people [27]. The scale was validated in Spanish and has a high confidence coefficient (0.81) and can therefore be appropriately used and applied in other institutions [23]. For this study, the Kogan scale scores were analyzed, and the results were categorized according to scores previously used: negative attitude from 34 to 118 points, low-level positive attitude from 119 to 146 points, middle-level positive attitude from 147 to 174 points, and high-level positive attitude from 175 to 204 points [27].

*Educational variables:* These variables were registered by each participant in a self-designed survey included in the Google Form. The variables and categories recorded were Faculty (Social Sciences and Communications, Law, Natural Resources and Veterinary Medicine, Health, Other faculties (Sciences, Economics and Business, Education, Engineering)), program (Psychology, Law, Veterinary medicine, Occupational therapy, Medical technology, Nursing, Physical Therapy, Speech Therapy, Nutrition and Dietetic, Other programs (Bachelor of Science, Social Work, Business Engineering, and Civil Engineering)), year (First, Second, Third, Fourth, Fifth, and Sixth year), subjects related to older people taken (Yes, No), and external courses related to older people taken (Yes, No).

*Sociodemographic variables*: These variables were also registered as factors associated with attitudes towards old age. These were registered by each participant in a self-designed survey included in the Google Form. The variables and categories considered were age (quartiles: 18–21/22–23/24–25/26–42 years), sex (male/female), nationality (Chilean/Foreigner), and ethnicity (Aimara, Quechua, Atacameño, Colla and Diaguita/Mapuche, Kawashqar, Yamana, and Yagan/Another ethnicity/none).

### 2.5. Statistical Analysis

The data were analyzed by means of SPSS 26.0 (IBM SPSS statistics, Chicago, IL, USA). Categories and variables were presented by frequency and percentage, while scalar variables were presented by mean and its corresponding confidence interval (95%). The distribution of data was measured through a Kolmogorov–Smirnov test, and homogeneity of variances was measured through a Levene’s test. Having a normal distribution and homogeneity of variances, parametric statistics were used. Firstly, prevalence of attitudes towards old age was presented, and secondly, a one-way ANOVA test was carried out to establish differences between the scores of Kogan’s test, in terms of socioeducational and sociodemographic variables. When a significant effect was evidenced, a Bonferroni post hoc analysis was performed, aiming to determine differences between groups. Level of significance *p* < 0.05.

## 3. Results

The final sample analyzed consisted of 515 students (64.3% women, average age 23.0 (±4.1)). Table 1 shows scores in Kogan’s test and perception towards old age in the university students studied. The average score on Kogan’s scale was 139.1 (16.6) points, and students generally perceived themselves with a low-level positive attitude (61.2%).

Table 2 shows the sociodemographic characteristics of the students. The one-way ANOVA performed evidenced significant differences in attitudes towards old age for the variables of age F(3;511) = 5.696, *p* = 0.001, sex F(1;511) = 6.038, *p* = 0.014, and nationality F(1;513) = 5.804, *p* = 0.016. A deeper analysis comparing pairs through a Bonferroni post hoc test asserts that older students (26–42 years old) scored higher on Kogan’s test when compared to other students (*p* < 0.05). Moreover, it was observed that women (*p* < 0.05) and Chileans (*p* < 0.05) achieved a higher score than men and foreigners, respectively.

Table 3 portrays educational variables analyzed in university students. A one-way ANOVA adduces significant differences in attitude towards old age measured through Kogan’s scale for the variables faculty F(4;510) = 3.368, *p* = 0.010, program F(9;505) = 4.606, *p* < 0.0001, year F(4;510) = 3.022, *p* = 0.018, and external courses related to older people F(1;513) = 8.650, *p* = 0.003. A deeper analysis comparing pairs through a Bonferroni post hoc test attests that students from programs such as law, speech therapy, and occupational therapy obtained higher scores than the rest of the students (*p* < 0.05). Furthermore, students in their fifth or sixth year of the program achieved higher scores than students in lower years (*p* < 0.05). Additionally, students who indicated having taken external courses related to older people reached a higher score on Kogan’s test than those who had not taken any external courses related to older people (*p* < 0.05).

## 4. Discussion

### 4.1. Main Findings of This Study

This study allowed us to discover students from a Chilean university’s attitudes towards older people according to sociodemographic and educational variables. The average score for positive attitude was 70.8 (±9.7), while the negative attitude score was 68.3 (±11.6). The total score was 139.1 (±16.6). The students perceived themselves with a generally low-level positive attitude towards old age (61.2%). Furthermore, it was evidenced that older university students (26–42 years old), women, and Chileans had a more positive attitude towards older people when compared to younger students, male students, and foreign students, respectively. Finally, it is suggested that students of law, speech therapy and occupational therapy; students in their final years of the programs; and those who had training in older people outside the university had a more positive attitude towards older people than their peers from other programs, from lower years of their programs, and who have not taken external courses about older people, respectively.

### 4.2. How Can the Results Be Interpreted in Perspective of Previous Studies?

Our results are consistent with previous articles that analyzed university students and that observed a trend towards a positive attitude [28]. Furthermore, these results are in line with international evidence suggesting that health sciences students and women have a more positive attitude towards aging compared to students from social sciences and humanities programs and men, respectively [28,29]. Likewise, students who acquire better knowledge in all aspects of aging through their education develop more-positive attitudes and interest in working with older adults [28,30].

This study aligns with the current tendency in which attitudes in students towards old age are neutral or positive, which also agrees with several international studies [31,32,33]. These attitudes depend on numerous factors, such as large numbers of hours in practices involving older people during their university training [34] or being in the last years of their programs [35], which can be caused by constant exposure and contact with older people in their university, family, and school activities [36]. Finally, a positive attitude towards old age can be related to interest in topics related to older adults and with four human strengths, such as love, kindness, gratitude, and humbleness [37].

Regarding older students with positive attitudes towards old age, there is a study ([38]) that agrees with this matter, in which a group of women from 20 to 30 years old presented a neutral attitude, while a group of older women presented a more positive attitude towards old age [9]. A possible explanation for this phenomenon could be that it is typically daughters who take care of their parents in the last stages of aging. Encinas et al. (2019) explained that female students have a more positive attitude towards older adults, and interestingly, the authors note that students who have close contact with older family members have a greater understanding that older adults need support and love, while students who do not have such contact consider older adults to be politically powerless [39]. Similarly, Rupp et al. (2005) observed that young men obtained significantly higher scores in ageism compared to older men and women [40]. It has been documented that stereotypes in young individuals can be an obstacle for the development of the knowledge and empathy needed for attending to older people [41].

Likewise, a cross-sectional study found that older people with negative beliefs about old age and aging showed a decline in their self-image in an interval of 8 years, while positive opinions predicted a better self-image [42]. These examples highlight the importance of our attitudes, as a society, towards aging, which is an important part of the circle of life.

### 4.3. Limitations

Like all studies, this research presents limitations. One of them is the impossibility of generalizing the results to students from other Chilean universities, since the study was only carried out in one university. It is possible that the method of recruitment (voluntary subjects from only one university) could have influenced the interpretation of the results obtained in this study. The sample was heterogeneous among university students. Most of the students belonged to health sciences careers and were in the first years of their studies; on the contrary, there was low participation of engineering sciences students or those who were in the last years of their programs. Additionally, self-report instruments were used, which could represent bias due to under- or over-self-evaluation of the variables studied. However, the use of questionnaires and online data collection have proven to be an acceptable method in cross-sectional studies, even more so at times when confinement due to COVID-19 makes it difficult to collect data in person [43].

Moreover, it is possible that some programs additionally deliver workshops related to aging or have older teachers, which could have influenced students’ attitudes towards older people. Finally, it should be noted that there is a large number and variety of instruments to measure attitudes towards old age, which may produce noncomparable results in different studies.

### 4.4. Contributions and Practical Implications

*Contributions to theory:* The population is aging rapidly, and university students must be aware of this demographic transition. In that line, increased education about the aging process could help reduce anxiety and stereotypes against the aged [44]. This study contributes to the emerging evidence provided by Latin American [19,45] and national [46] studies that analyze the attitude towards older people in university students. This study provides national information about attitudes towards older people from university students according to sociodemographic and educational variables, becoming the most current evidence that accounts for the attitude towards aging of Chilean university students.

*Practical implications:* This study generates evidence that helps support the generation of innovation strategies, especially in the creation of practical activities in universities, as well as technical colleges and other professional institutions. Specifically, it is suggested to design intervention plans in university students based on empathy [47] and positive education about aging and contact experiences [48], which could potentially be part of an international strategy to improve perceptions of older people and the aging process. Thus, there is evidence of interventions based on high-fidelity simulation carried out with nursing students, who underwent training for the development of attitudes towards older adults and empathy skills, who found an improvement in the scores obtained on Kogan’s scale and therefore improving the levels of empathy and positive attitudes towards old age [49]. Additional research using more-rigorous designs to examine the effects of interventions is strongly recommended [47,50]. Notably, some research concludes that some students would need to have targeted education to help them be more positive with older people, as it would be the beginning of further treatment as professionals [51].

Contents and topics related to aging and competences oriented towards the good treatment of the elderly should be kept in mind when redesigning and making changes to the curriculum of several university programs to foster well-rounded education that includes interaction with older people and their environments. All of this should be conducted with the aim of minimizing negative and ageist attitudes from the students. In this line, it is suggested to use the recommendations given in the global report on ageism of the World Health Organization as a framework [52].

Evidence suggests that the quality of care provided to older people is related to the attitudes of health professionals, indicating that health educators should adopt effective strategies to increase and promote students’ positive attitudes about older people [53,54]. Thus, it is important that the curriculum of programs related to health sciences should incorporate a formative process that allows the students to get familiar with the effects of aging, providing them with well-rounded education in terms of geriatrics and gerontology, which could prevent them from holding negative stereotypes, especially ageism [55,56]. A positive attitude from healthcare professionals towards older adults will encourage people of this age group to continue with their activities, while a negative attitude can greatly impact the quality of healthcare services, as well as in the quality of life of older adult patients. We should aim for the prevention of certain dismissive attitudes from older adults towards themselves, since they negatively affect their self-esteem. Positive attitudes, understood as a predisposition to perceive and behave in a certain manner, can be promoted by a high degree of empathy. In contrast, a negative attitude can be caused by rejection or avoidance of the subject [16].

### 4.5. Future Lines of Research

Attitudes towards old age should continue to be researched to understand which areas of undergraduate and postgraduate programs would need adjustments, since it is paramount to prepare students to deal with the needs of older adults, an age group that is increasingly growing. It is also important to educate students without ageist attitudes in their initial training, reinforcing these ideas throughout the entire circle of life. Researchers are encouraged to delve deeply into the factors that could model university students’ attitudes towards the elderly. Thus, whether having lived with grandparents, having contact with older people, or self-perception of health could impact university students’ attitudes towards older people can be studied.

## 5. Conclusions

The participants of the study who presented a negative perception towards older people fit into a specific sociodemographic and educational profile. These results are relevant, since the attitude towards old age can influence the initial training of future professionals, therefore affecting their disposition to dealing with older people.

More studies on this area are needed in order to create strategies that allow for the decrease in stereotypes towards aging and older adults.

All in all, the incorporation of activities related to older people in the curriculum of university programs can help promote a positive perception of the later stages of the circle of life, thus fighting negative stereotypes in the community towards older adults. Young people’s perception of aging would affect the treatment and incorporation of the older people in society and the adaptation of policies in this age group.

## Figures and Tables

**Table 1:** Kogan’s scale scores and attitudes towards old age.

Variables	
*Kogan’s scale score*	M(SD)
Total score	139.1 (16.6)
Positive statements score	70.8 (9.7)
Negative statements score	68.3 (11.6)
*Attitude towards old age*	N (%)
Negative attitude	46 (8.9%)
Positive attitude, low level	315 (61.2%)
Positive attitude, middle level	144 (28.0%)
Positive attitude, high level	10 (1.9%)

**Table 2:** Sociodemographic characteristics.

		Total Score	One-Way ANOVA
Variables	N (%)	Mean [95% CI]	*p*-Value
Age (quartiles)			
*Quartile 1 (18–21 years)*	166 (32.2%)	136.63 [134.12; 139.14] a	0.001
*Quartile 2 (22–23 years)*	143 (27.8%)	138.43 [135.66; 141.21] a	
*Quartile 3 (24–25 years)*	90 (17.5%)	137.82 [134.75; 140.90] a	
*Quartile 4 (26–42 years)*	116 (22.5%)	144.47 [141.35; 147.58] b	
Sex			
*Male*	183 (35.7%)	136.72 [134.49; 138.95]	0.014
*Female*	330 (64.3%)	140.43 [138.59; 142.27]	
Nationality			
*Chileans*	502 (97.5%)	139.39 [137.93; 140.84]	0.016
*Foreigners*	13 (2.5%)	128.23 [122.27; 134.19]	
Ethnicity			
*Aimara, quechua, atacameño, colla, and diaguita*	45 (8.7%)	137.73 [132.50; 142.97]	0.479
*Mapuche, kawashqar, yamana, and yagan*	56 (10.9%)	139.80 [135.75; 143.86]	
*Another ethnicity*	6 (1.2%)	129.50 [122.45; 136.55]	
*None*	408 (79.2%)	139.30 [137.67; 140.93]	

Statistical analysis carried out through one-way ANOVA. ab means that within same line signal, there are statistically significant differences between groups. (One-way ANOVA and Bonferroni post hoc test).

**Table 3:** Educational variables.

		Total Score	One-Way ANOVA
Variables	N (%)	Mean [95% CI]	*p*-Value
Faculty			
*Social Sciences and Communications*	14 (2.7%)	142.21 [134.38; 150.05] ab	0.010
*Law*	12 (2.3%)	154.17 [144.55; 163.79] b	
*Natural Resources and Veterinary Medicine*	13 (2.5%)	132.31 [119.60; 145.02] a	
*Health*	466 (90.5%)	138.74 [137.25; 140.24] ab	
*Other faculties (Sciences, Economics and Business, Education, Engineering)*	10 (1.9%)	142.40 [132.50; 152.30] ab	
Program			
*Psychology*	12 (2.3%)	141.75 [132.47; 151.03] abc	<0.0001
*Law*	12 (2.3%)	154.17 [144.55; 163.79] c	
*Veterinary Medicine*	13 (2.5%)	132.31 [119.60; 145.02] ab	
*Occupational Therapy*	63 (12.2%)	144.05 [139.94; 148.16] bc	
*Medical Technology*	16 (3.1%)	126.56 [116.67; 136.46] a	
*Nursing*	63 (12.2%)	140.51 [136.19; 144.83] abc	
*Physical Therapy*	296 (57.5%)	138.54 [136.79; 140.29] abc	
*Speech Therapy*	11 (2.1%)	144.00 [128.75; 159.25] bc	
*Nutrition and Dietetics*	16 (3.1%)	126.00 [120.00; 132.00] a	
*Other programs (Bachelor of Science, Social Work, Business Engineering, and Civil Engineering)*	13 (2.5%)	139.00 [127.89; 150.11] abc	
Year			
*First year*	92 (17.9%)	135.30 [131.99; 138,62] a	0.018
*Second year*	80 (15.5%)	138.95 [135.03; 142.87] ab	
*Third year*	136 (26.4%)	138.46 [153.60; 141.32] ab	
*Fourth year*	145 (28.2%)	139.91 [137.38; 142.44] ab	
*Fifth and sixth year*	62 (12.0%)	144.47 [140.17; 148.76] b	
Did you have any subjects related to older people in your program?			
*Yes*	369 (71.7%)	139.6 [137.93; 141.27]	0.280
*No*	146 (28.3%)	137.85 [135.04; 140.66]	
Did you take any external courses related to older people?			
*Yes*	247 (48.0%)	141.32 [139.29; 143.36]	0.003
*No*	268 (52.0%)	137.06 [135.06; 139.06]	

Statistical analysis carried out through one-way ANOVA. abc means that within same line signal, there are statistically significant differences between groups. (One-way ANOVA and Bonferroni post hoc test).

## Data Availability

Data will be made available upon request.
